# Water fluoridation, functional dentition and tooth loss in Brazilian adults: a multilevel analysis of SB Brasil 2023

**DOI:** 10.1590/1980-549720260031.supl.1

**Published:** 2026-08-17

**Authors:** Paulo Roberto Barbato, José Leopoldo Ferreira Antunes, Paulo Frazao, Marco Aurélio Peres

**Affiliations:** I Universidade Federal de Santa Catarina, Department of Speech-Language Pathology - Florianópolis (SC), Brazil.; II Universidade de São Paulo, School of Public Health - São Paulo (SP), Brazil.; III National Dental Research Institute Singapore, National Dental Centre - Singapore.; IV Oral Health Academic Clinical Programme, Duke-NUS Medical School - Singapore.; V Health Services and Population Health Research Programme, Duke-NUS Medical School - Singapore.

**Keywords:** Tooth loss, Surveys, Multilevel modelling, Fluoridated water, Dental care

## Abstract

**Objective:**

To evaluate the association between water fluoridation and the maintenance of functional dentition (≥20 teeth) and tooth loss in Brazilian adults aged 35 to 44, considering individual and contextual factors.

**Methods:**

This study employed a cross-sectional design using data from SB Brasil 2023 (n=9,019). The main exposure was the availability of fluoridated water in municipalities, obtained from national sanitation information systems. The outcomes analyzed were (i) functional dentition (binary) and (ii) number of missing teeth (count). To reduce confounding bias, inverse probability weighting was used to balance individual (age, sex, education, and income) and contextual (municipal development index and dental service coverage) covariates. The analysis was performed using multilevel Poisson models for functional dentition and multilevel negative binomial models for missing teeth. The robustness of the results was assessed using E-values, estimating sensitivity to potential unmeasured confounders.

**Results:**

After adjustment, fluoridation showed no significant association with functional dentition (PR 1.24; 95%CI 0.91-1.68). However, it was significantly associated with a lower rate of missing teeth (RR 1.30; 95%CI 1.01-1.67). Sensitivity analysis (E-value) indicated high susceptibility to unmeasured confounders for the count outcome.

**Conclusion:**

In the context of widespread use of fluoridated dentifrice, water fluoridation demonstrated a significant protective effect against tooth loss in adults, although it was totally or partially confounded by socioeconomic variables. These findings reinforce the importance of universal fluoridation policies, which should be combined with strategies targeting vulnerable groups, aiming to reduce inequalities and promote oral health in the adult population.

## INTRODUCTION

Tooth loss remains one of the most sensitive and cumulative markers of health inequalities across the course of life^1^ ^,^ ^2^. Although preventable, it continues to be highly prevalent among adults and older people, especially in middle-income countries, reflecting the impact of untreated oral diseases, unequal access to dental services, and persistent social determinants^3^ ^,^ ^4^ ^,^ ^5^.

In this study, we analyzed two conceptually distinct outcomes:

1. Functional dentition: generally defined as the presence of at least 20 natural teeth associated with masticatory ability, adequate nutrition, and psychosocial well-being^6^ ^,^ ^7^, contributing to quality of life, active aging, and a reduced risk of functional decline and premature mortality^8^;
2. Number of missing teeth: cumulative outcome of an incremental and progressive process.

The former represents the quality of the dentition regarding functional performance; the latter, a simple quantitative measure.

Over the last two decades, Brazil has expanded access to oral health care, particularly through the integration of oral health teams (ESB) into the Family Health Strategy and the expansion of population-based preventive policies^9^ ^,^ ^10^. Evidence shows that municipalities with higher ESB coverage exhibit lower rates of tooth loss and greater retention of functional dentition, even after controlling for individual and contextual factors^11^ ^,^ ^12^. However, regional inequalities, as well as disparities based on income, education, and race/color, persist, indicating that access to and use of services remain strongly determined by structural factors^13^.

Among the most effective and equitable preventive measures, the fluoridation of public water supplies - a policy covering approximately 76% of the Brazilian population - and the widespread use of fluoride toothpaste stand out. Both measures have been expanded since the 1990s^10^.

National and international evidence demonstrates significant reductions in dental caries, the main cause of premature tooth loss, with water fluoridation offering a greater protective effect among lower-income groups^14^ ^,^ ^15^. Although a natural experiment in Brazil confirmed that water fluoridation is associated with reduced caries and tooth loss in adults^16^, the impact of fluoridation on the dentition of Brazilian adults, in the context of widespread fluoride toothpaste use, remains under-investigated. Thus, combining universal policies (fluoridation) with strategies to expand oral health coverage within primary health care is crucial for promoting equity and maintaining functional dentition among Brazilian adults. While the expansion and refinement of these policies remain incomplete ^17^ ^,^ ^18^, investigating their potential effects is significant for scientific knowledge.

Given the complexity of the social and structural determinants of tooth loss, traditional approaches may underestimate causal effects due to residual confounding and the hierarchical structure of the data^19^. The combined use of multilevel models and inverse probability of treatment weighting (IPTW) allows for the estimation of associations between individual and contextual factors and oral health outcomes^20^ ^,^ ^21^. Furthermore, E-value analysis quantifies sensitivity to residual bias, thereby strengthening causal inference in observational studies^22^.

The primary objective of this study was to evaluate the association between water fluoridation and the maintenance of functional dentition and tooth loss among Brazilian adults aged 35 to 44, while rigorously controlling for individual and contextual factors using multilevel models and IPTW adjustment, and quantifying the robustness of the associations against unmeasured confounders. The main hypothesis is that water fluoridation, acting as a population-level protective factor against dental caries, increases the likelihood of retaining functional dentition and reduces the number of lost teeth.

## METHODS

This is a population-based cross-sectional study using data from the 2023 National Oral Health Survey (SB Brasil). The study included the 35-44 age group (9,019 participants). The selection of this age group follows World Health Organization (WHO) recommendations, enabling comparability with national and international studies, including previous editions of SB Brasil. A total of 391 municipalities were included across all age groups investigated, representing both state capitals and municipalities in the interior of the 27 federative units. For adults, the data refer to residents of 352 municipalities. Details regarding sampling and fieldwork are available in the survey’s Final Report^23^. Examining dentists underwent theoretical-practical training and calibration, being deemed qualified upon achieving adequate agreement^23^.

This study used two outcomes. The first was functional dentition, constructed on the basis of the “lost” variable from the original dataset, adjusted to include teeth lost for other reasons and subtracted from the total number of teeth expected in adults, with results dichotomized into “presence of functional dentition” (20 or more teeth) and “absence of functional dentition” (fewer than 20 or no teeth). The second outcome was the number of teeth lost due to caries, based on the original “lost” variable.

The primary exposure was “availability of fluoridated water” treated as a binary variable (yes/no), measured at the contextual level, and derived from the National Sanitation Information System^24^ and the Information System for Drinking Water Quality Surveillance (2022-2023)^25^. To avoid classification errors, sanitation data for 2023 were compared with data from 2022 and 2021, as well as with water surveillance data for 2023 and 2022. A municipality’s public water supply was classified as fluoridated if the information was consistent across at least one of the years analyzed in both data sources, or consistent across more than one of the years analyzed in a single data source. The remaining municipalities were classified as non-fluoridated.

The individual-level covariates included were the following: sex, race/color, age group (35-39 and 40-44 years), years of schooling (13 or more, 10-12, and 9 or less, per capita income (in tertiles), time since the last dental visit (“utilization_service” in the original dataset), and the type of service used (“type_service_utilized” in the original dataset). At the contextual level, the estimated population coverage of oral health services within the Unified Health System (SUS) primary care network was also included (proportion of inhabitants potentially covered by ESB, categorized as 50-100% and <50%, according to Ministry of Health data from December 2023)^26^. The FIRJAN municipal development index (IFDM) was also included. This index, which ranges from 0 to 1, was developed by the Federation of Industries of the State of Rio de Janeiro to measure the socioeconomic development of Brazilian municipalities, taking into account three areas, i.e., employment and income, health, and education^27^. Municipalities are classified as having high (0.8 or higher), moderate (0.6-0.79), low (0.4-0.59), or critical (up to 0.39) development, according to the classification adopted by Firjan. The data refer to the 2023 base year (2025 edition).

**Figure 1.**
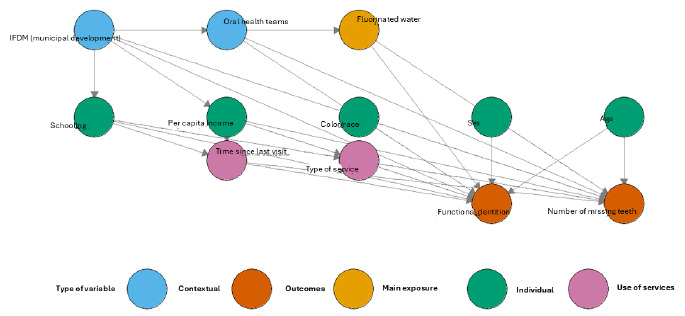
Directed acyclic graph for tooth loss in adults. SB Brasil 2023.

Source: Prepared by the authors. SB Brasil 2023 data.

A directed acyclic graph (DAG) for tooth loss (Figure 1) was constructed using the DAGitty package for R. The DAG aims to explain the causal relationships between the main exposure, the covariates, and the outcomes. It also illustrates the hierarchical structure of the data, with individuals nested within municipalities. Water fluoridation is expected to increase the likelihood of individuals having functional dentition and to reduce the number of lost teeth. However, as this is not an isolated relationship, the IFDM is included; it is an important contextual confounder, given that more developed municipalities are better positioned to maintain water fluoridation and public dental services. Such conditions reflect the inhabitants’ years of schooling and income, which in turn influence the outcomes.

To estimate the association with fluoridation, it is necessary to adjust for the IFDM and ESB coverage. The DAG also incorporates proximal individual-level variables. Age, a strong predictor of tooth loss, reflects the cumulative experience of disease over the life course. Years of schooling and income act as fundamental determinants, influencing health outcomes and behaviors. Individuals with higher education and income are expected to have greater access to information and health-related resources, seeking dental appointments more frequently and utilizing services that offer timely preventive and restorative care. Time since the last dental visit and type of service were treated as confounders, as they potentially precede the exposure and share common causes (socioeconomic status), without being considered mediators of the relationship between fluoridation and tooth loss. Failure to adjust for these variables could lead to residual confounding bias, given that they do not lie on the causal pathway between fluoridation and tooth loss. Adjusting for these factors is necessary to avoid biased estimates, without over-adjustment, thereby reflecting the direct effect of fluoridation independent of individual patterns of service utilization.

The statistical analysis was organized as follows: descriptive analysis weighted by sample weights, multilevel modeling for each outcome, and sensitivity analysis. All analyses were performed using StataNow software, version 19.5. The complex sampling design of the SB Brasil survey was accounted for in the descriptive analyses using sample weights (svy command).

The IPTW method was employed to adjust for covariate imbalance between those exposed and unexposed to water fluoridation. Propensity scores were estimated via logistic regression, and IPTW weights were calculated as the inverse of the probability of receiving the actually observed treatment. Covariate balance between exposure groups was assessed using standardized mean differences (SMD); balance was considered satisfactory when the SMD for all covariates was <0.10 in the weighted sample.

For the multilevel models using IPTW, stabilized IPTW weights were used as probability weights (*pweights*) without incorporating complex sampling weights, as the primary objective was to balance groups for determining association.

The regression analyses used a two-level multilevel model structure with block entry. For “absence of functional dentition”, multilevel Poisson regression with robust variance was used; effect was measured by the prevalence ratio (PR). For “number of missing teeth”, multilevel negative binomial regression was used to model the count outcome, which exhibited potential overdispersion. Rate ratios (RR) were calculated as the measure of association, and the overdispersion parameter was reported to assess model fit.

The models followed this variable inclusion order: M0 (null model) - random intercept only - , M1 (main exposure), M2 (main exposure + individual-level covariates), and M3 (contextual + individual-level exposures).

To assess random effects, the magnitude of variation between municipalities was presented via the intercept variance. The itraclass correlation coefficient (ICC) was calculated for the Poisson regression analysis to quantify the proportion of total outcome variance attributable to the contextual level. A likelihood-ratio test was used to compare each model with the preceding one, determining whether the inclusion of each new block of variables resulted in a significantly better model fit.

Completeness of contextual and individual-level variables was ensured across all regression models. Any missing data were handled via listwise deletion. Because of the listwise deletion of participants with incomplete individual-level data, the final sample for the functional dentition model consisted of 6,286 individuals across 298 municipalities; For the model regarding the number of missing teeth, the sample consisted of 6,279 individuals across the same 298 municipalities. Details of the sample flow are presented in the supplementary material (Figure S1 and Table S1).

To assess the potential impact of unmeasured confounding, a sensitivity analysis using E-values was performed, considering the association between the primary exposure and each outcome^28^. The E-value represents the minimum strength an unmeasured confounder would need to possess to nullify the observed association between fluoride availability and the outcome.

The SB Brasil 2023 project was approved in July 2021 (Opinion No. 4.823.054) by the National Research Ethics Commission (Conep), in accordance with Resolution No. 466/2012 of the National Health Council.

**Table 1.** Distribution of the study population (adults aged 35-44 years) according to outcomes and individual and contextual variables, proportion of functional dentition, and mean number of teeth lost. SB Brasil 2023.

Outcome	% (95%CI)*		
Functional dentition			
Yes	92.7 (90.7-94.4)		
No	7.3 (5.6-9.3)		
**Number of missing teeth**	**Mean (95%CI)**	**Standard deviation**	
	3.5 (3.0-3.9)	5.5	
**Individual variables (n=9,019)**	% (95%CI)*	**With functional dentition**	**Missing teeth**
		**% (95%CI)**	**Mean (95%CI)**
Sex			
Male	33.7 (31.2-36.3)	92.2 (87.8-95.0)	3.6 (2.8-4.5)
Female	66.3 (63.7-68.8)	93.1 (90.9-94.7)	3.4 (3.0-3.7)
Color/race			
White	42.2 (38.3-46.3)	93.9 (90.8-96.0)	2.8 (2.3-3.3)
Black	13.5 (11.3-16.1)	92.5 (88.4-95.3)	4.3 (3.5-5.2)
Asian	1.4 (0.9-2.1)	93.6 (84.5-97.5)	4.3 (2.8-5.8)
Brown	42.6 (38.7-46.7)	91.9 (88.6-94.3)	3.8 (3.1-4.5)
Indigenous	0.3 (0.2-0.6)	92.7 (78.4-97.8)	4.7 (2.7-6.8)
Age group (years)			
35-39	49.3 (46.0-52.5)	96.1 (94.1-97.4)	2.6 (2.2-3.0)
40-44	50.7 (47.5-54.0)	89.5 (85.9-92.3)	4.3 (3.6-5.0)
Years of schooling			
13 or more	23.1 (19.6-27.1)	99.4 (99.0-99.7)	1.1 (0.9-1.3)
10 to 12	48.5 (44.6-52.5)	95.2 (93.4-96.6)	2.9 (2.5-3.2)
Up to 9	28.4 (25.2-31.8)	84.4 (79.7-88.1)	6.2 (5.3-7.1)
Per capita income (tertiles)			
1st	29.5 (26.0-33.4)	87.7 (81.6-92.0)	5.2 (4.1-6.3)
2nd	41.2 (36.5-46.0)	92.8 (88.1-95.7)	3.3 (2.7-3.9)
3rd	29.3 (24.0-35.1)	97.6 (95.9-98.7)	2.0 (1.6-2.5)
Last dental visit (years)			
Never a visit	0.9 (0.5-1.7)	90.6 (65.1-98.0)	4.8 (3.0-6.7)
Up to 1	51.6 (48.3-55.0)	94.1 (91.8-95.7)	3.0 (2.6-3.4)
>1-2	19.8 (17.8-22.0)	91.9 (85.1-95.7)	3.8 (2.7-4.9)
>2-3	12.6 (10.4-15.1)	89.9 (81.8-94.6)	3.7 (2.7-4.7)
>3	15.1 (12.9-17.7)	90.4 (86.2-93.4)	4.6 (3.7-5.5)
Place of visit			
Never a visit	0.9 (0.5-1.7)	90.6 (65.1-98.0)	4.8 (3.0-6.7)
Public service	42.8 (37.5-48.4)	92.1 (89.9-93.9)	3.9 (3.4-4.5)
Private service	47.0 (42.5-51.5)	91.9 (87.8-94.7)	3.3 (2.6-4.0)
Health insurance/plan	8.6 (6.6-11.2)	97.7 (94.5-99.1)	2.4 (1.7-3.1)
Other	0.7 (0.3-1.6)	91.8 (69.2-98.2)	4.0 (1.3-6.7)
Contextual variables (n=352)	% (95%CI)		
IFDM			
High	24.4 (18.4-31.7)		
Moderate	50.8 (42.8-58.7)		
Low	23.4 (19.0-28.5)		
Critical	1.4 (0.6-3.5)		
Availability of fluoridated water			
Yes	69.7 (64.3-74.5)		
No	30.3 (25.5-35.7)		
Oral health coverage in PHC			
50-100%	54.4 (46. 5-62.0)		
<50%	45.6 (38.0-53.5)		

*Weighted values.

CI: confidence interval; IFDM: FIRJAN municipal development index; PHC: primary health care.

Source: Prepared by the authors. SB Brasil 2023 data.

## Data availability statement:

The complete dataset supporting the results of this study is available upon request to the corresponding author. The dataset is not publicly available due to the database integration performed by the authors.

## RESULTS

The sample included 9,019 Brazilian adults aged 35 to 44, predominantly women (66.3%) and individuals of mixed race (“pardos”, 42.6%). The majority possessed functional dentition (92.7%) and had an average of 3.5 missing teeth (SD=5.5). Almost half had between 10 and 12 years of schooling, and 23.1% were currently attending or had completed higher education. Most fell within the per capita income range of 0.36 to 0.75 times the minimum wage in effect for the greater part of 2023 (R$ 1,320). More than half had visited a dentist in the past year, with 42.8% using public services. At the municipal level, 69.7% resided in areas with water fluoridation, and 54.4% in locations with ESB coverage exceeding 50%. Municipalities with moderate socioeconomic development predominated (50.8%). These results reflect a population with good average dental conditions, yet one still marked by structural inequalities regarding income, education, and access to services and water fluoridation.

Results regarding the absence of functional dentition are presented in Table 2. In the crude analysis (Model 1), the absence of water fluoridation was associated with a higher prevalence of the outcome (PR 1.72; 95%CI 1.22-2.43). In the analysis adjusted for individual factors (Model 2), the absence of fluoridation was associated with a PR of 1.40 (95%CI 1.03-1.91). In the full model (Model 3), PR was lowered to 1.24 (95%CI 0.91-1.68), with a confidence interval that included the null value (1.00). Regarding the contextual aspect, significant inter-municipal variability was observed in the null model, with an ICC indicating that 38% of the total variance in the prevalence of the absence of functional dentition is attributable to differences between municipalities. This variability decreased upon the inclusion of covariates, reaching an ICC of 0.217 in the fully adjusted model, suggesting that approximately 43% of the initial contextual variance was explained by the variables included in the final model.

**Table 2.** Multilevel robust variance Poisson regression with inverse probability of treatment weighting for the absence of functional dentition in adults (35-44 years) - n=298 municipalities and 6,286 participants. SB Brasil 2023.

Fixed effects	Null model	Model 1		Model 2		Model 3	
		PR (95%CI)*	p-value	PR (95%CI)	p-value	PR (95%CI)	p-value
Contextual factors							
Fluorinated water							
Yes		REF.	0.002	REF.	0.031	REF.	0.141
No		1.72 (1.22-2.43)		1.40 (1.03-1.91)		1.24 (0.91-1.68)	
Oral health coverage in PHC^†^							
50-100%						REF.	0.365
<50%						0.82 (0.59-1.14)	
IFDM							
High						REF.	0.049
Moderate						1.48 (0.89-2.46)	
Low						1.76 (0.97-3.19)	
Critical						2.98 (1.09-8.19)	
Individual factors							
Sex							
Female				REF.	0.634	REF.	0.632
Male				1.11 (0.72-1.71)		1.11 (0.72-1.71)	
Color/race							
White				REF.	0.337	REF.	0.250
Black				0.84 (0.55-1.29)		0.81 (0.52-1.24)	
Asian				1.28 (0.75-2.18)		1.24 (0.72-212)	
Brown				0.87 (0.66-1.15)		0.84 (0.64-1.11)	
Indigenous				0.89 (0.26-3.01)		0.86 (0.26-2.90)	
Age group (years)							
35-39				REF.	<0.001	REF.	<0.001
40-44				2.31 (1.78-2.99)		2.33 (1.80-3.01)	
Years of schooling							
13 or more				REF.	< 0.001	REF.	<0.001
10 to 12				2.34 (1.17-4.69)		2.37 (1.18-4.75)	
Up to 9				5.77 (2.61-12.76))		5.85 (2.64-12.93)	
Per capita income (tertiles)							
1st				REF.	0.062	REF.	0.094
2nd				0.80 (0.57-1.13)		0.83 (0.59-1.17)	
3rd				0.64 (0.41-0.97)		0.66 (0.43-1.02)	
Last dental visit (years)							
Never a visit				REF.	0.443	REF.	0.439
Up to 1				0.21 (0.02-2.21)		0.22 (0.02-2.29)	
>1-2				0.20 (0.02-2.29)		0.21 (0.02-2.35)	
>2-3				0.17 (0.02-1.73)		0.17 (0.02-1.76)	
>3				0.26 (0.03-2.63)		0.27 (0.03-2.74)	
Place of visit							
Never a visit				REF.	0.280	REF.	0.351
Public service				6.62 (0.75-51.74)		6.39 (0.82-50.03)	
Private service				6.81 (0.88-52.49)		6.67 (0.87-51.47)	
Health insurance/plan				4.69 (0.59-37.17)		4.58 (0.58-36.29)	
Other				-		-	
Random effects							
Variance between municipalities	0.614	0.538		0.349		0.278	
ICC	0.380	0.350		0.259		0.217	
Likelihood-ratio test		p=0.013		p<0.001		p=0.058	

*Prevalence ratio and 95% confidence interval; ^†^oral health coverage in primary health care. PR: prevalence ratio; CI: confidence interval; OH: oral health; PHC: primary health care; IFDM: FIRJAN municipal development index; IPTW: inverse probability of treatment weighting; ICC: intraclass correlation coefficient.

Source: Prepared by the authors. SB Brasil 2023 data.

Table 3 presents the results of the multilevel negative binomial regression for the number of lost teeth. For this outcome, water fluoridation was associated with a lower rate of tooth loss (RR 1.30; 95%CI 1.01-1.67) in the full model, indicating that the absence of fluoridation increases the tooth loss count by 30%.

**Table 3.** Multilevel negative binomial regression with inverse probability of treatment weighting for the number of lost teeth in adults (35-44 years) - n=298 municipalities and 6,279 participants. SB Brasil 2023.

Variables	Fixed effects							
	Model 0		Model 1		Model 2		Model 3	
	RR (95%CI)*	p-value	RR (95%CI)	p-value	RR (95%CI)	p-value	RR (95%CI)	p-value
Fluorinated water								
Yes			REF.	0.003	REF.	0.004	REF.	0.034
No			1.44 (1.13-1.83)		1.34 (1.09-1.64)		1.30 (1.01-1.67)	
Oral health coverage in PHC^†^								
50-100%							REF.	0.557
<50%							1.05 (0.83-1.32)	
IFDM								
High							REF.	0.607
Moderate							1.28 (0.91-1.80)	
Low							1.27 (0.83-1.96)	
Critical							1.08 (0.34-3.48)	
Sex								
Female					REF.	0.246	REF.	0.246
Male					1.10 (0.95-1.28)		1.10 (0.95-1.28)	
Color/race								
White					REF.	0.046	REF.	0.050
Black					1.13 (0.94-1.37)		1.13 (0.94-1.36)	
Asian					1.24 (0.88-1.75)		1.24 (0.88-1.75)	
Brown					1.11 (1.00-1.24)		1.11 (0.99-1.24)	
Indigenous					1.58 (1.04-2.39)		1.58 (1.04-2.38)	
Age group (years)								
35-39					REF.	<0.001	REF.	<0.001
40-44					1.55 (1.40-1.71)		1.55 (1.40-1.71)	
Years of schooling								
13 or more					REF.	<0.001	REF.	<0.001
10 to 12					1.80 (1.52-2.13)		1.81 (1.53-2.14)	
Up to 9					2.74 (2.18-3.45)		2.75 (2.18-3.45)	
Per capita income (tertiles)								
1st					REF.	<0.001	REF.	<0.001
2nd					0.89 (0.80-0.99)		0.89 (0.80-0.99)	
3rd					0.68 (0.59-0.78)		0.68 (0.60-0.78)	
Last dental visit (years)								
Never a visit					REF.	0.152	REF.	0.154
Up to 1					1.87 (0.64-5.48)		1.88 (0.65-5.49)	
>1-2					1.89 (0.62-5.69)		1.89 (0.63-5.69)	
>2-3					1.83 (0.63-5.31)		1.84 (0.64-5.32)	
>3					2.13 (0.69-6.54)		2.14 (0.70-6.55)	
Place of visit								
Never a visit					REF.	0.663	REF.	0.666
Public service					0.73 (0.47-1.11)		0.72 (0.47-1.11)	
Private service					0.73 (0.47-1.13)		0.73 (0.47-1.11)	
Health insurance/plan					0.64 (0.41-1.01)		0.64 (0.41-1.00)	
Other					-		-	
Random effects								
Variance between municipalities	0.548		0.503		0.479		0.448	
Overdispersion parameter	0.661		0.662		0.590		0.591	
Likelihood ratio test			p<0.001		p<0.001		p<0.001	

*Rate ratio and 95% confidence interval; ^†^Oral health coverage in primary health care.

RR: rate ratio; CI: confidence interval; SB: oral health; PHC: primary health care; IFDM: FIRJAN municipal development index.

Source: Prepared by the authors. SB Brasil 2023 data.

Analysis of random effects revealed that 18.2% of the initial inter-municipal variance was explained by the covariates in the full model. The overdispersion parameter (0.591) confirmed the suitability of the negative binomial model, and likelihood-ratio tests indicated a significant improvement in model fit with the progressive inclusion of covariates (p<0.001).

Covariate balance between those exposed and unexposed to fluoridated water, both before and after applying IPTW weights, was assessed using SMD. Prior to weighting, some variables showed imbalance; after applying IPTW weights, all covariates exhibited SMDs below 0.10 (with the majority below 0.05), demonstrating adequate balance between the groups. These results indicate that the IPTW procedure was effective, creating comparable groups regarding measured characteristics and allowing for a less biased estimate of the fluoridation effect.

Sensitivity analysis using E-values ​​was conducted to evaluate the potential influence of unmeasured confounders. For the functional dentition outcome, the E-value for the confidence interval limit was 1.00, a result consistent with the lack of statistical significance observed in the main analysis. For the number of lost teeth, the E-value for the lower limit of the confidence interval was 1.11, indicating high susceptibility to residual confounding, where an unmeasured factor of small magnitude could nullify the observed association. Therefore, a causal interpretation must be cautious.

## DISCUSSION

Among Brazilian adults aged 35 to 44, the availability of fluoridated water was associated with fewer lost teeth but not with the maintenance of functional dentition after adjustment for individual and contextual factors. The association regarding the tooth count outcome showed sensitivity to residual confounding, suggesting the need for cautious interpretation. Overall, the findings are consistent with a population-level benefit of fluoridation regarding incremental tooth loss, though they do not support a strong causal inference. This association, interpreted as the estimated effect of the exposure context, reinforces the importance of public oral health policies, demonstrating that population-level interventions like water fluoridation continue to have a measurable impact on preserving functional teeth.

Tooth loss among Brazilian adults has gradually declined in recent decades as a result of prevention and treatment policies^9^. Even amidst the widespread use of fluoride toothpaste and socioeconomic inequalities, water fluoridation demonstrated a protective effect against tooth loss, similar to that described by Roberto et al.^29^.

Regarding functional dentition, although an association with fluoridation was observed in simple analyses and those adjusted for individual characteristics, statistical significance was lost after IFDM adjustment. This suggests that municipal development may influence the conditions necessary for implementing water fluoridation and maintaining public dental service networks. The high prevalence of functional dentition in the sample (92.7%) limits the ability to identify factors associated with its absence, which may explain why the association with fluoridation lost significance after contextual adjustment.

The potential of universal policies like fluoridation can be maximized or limited by local social dynamics. In highly developed areas, two trends may be observed: the overall estimated effect tends to diminish over time, and the protective association tends to be more pronounced among socially vulnerable groups compared to more affluent ones^14^ ^,^ ^15^.

The results reinforce the need to maintain and expand water fluoridation to prevent population-level tooth loss, particularly in contexts of social inequality. The estimated effect of fluoridation remained consistent even after adjusting for ESB coverage, suggesting complementarity between the universal strategy (fluoridation) and the care-based strategy (ESB). To enhance equity, the expansion of fluoridation must be accompanied by the strengthening of primary health care in municipalities with lower socioeconomic development.

Interpreting adjusted coefficients from multilevel tables in isolation, known as the Table 2 fallacy, can lead to erroneous conclusions regarding the causal association of individual or contextual variables^30^. The observed associations with fluoridation should be understood as model-conditioned average estimates, simultaneously accounting for interactions between individual and contextual factors rather than as the factor’s absolute, independent effect. The use of IPTW combined with multilevel models and E-value sensitivity analysis strengthens the inference.

The cross-sectional design precludes establishing a temporal relationship between outcomes and exposure; however, the fact that water fluoridation is a long-standing policy and tooth loss is a cumulative process mitigates this limitation. As discussed by Frazão and Spencer^31^, observational studies on water fluoridation face significant methodological challenges, such as the difficulty of determining exposure duration and controlling for fluoride concentration fluctuations over time, which may result in attenuated estimates or a degree of uncertainty. Nevertheless, comparing exposed and unexposed groups using official data sources represents the best available evidence for assessing differences resulting from fluoridation exposure across various study designs and population-level analyses.

Another limitation is the ecological nature of the exposure (fluoridation), which restricts inferences regarding direct individual effects; it does not allow for the identification of specific individuals who were actually exposed to or benefited by the policy, nor does it fully rule out the effects of inter-municipal migration or the “halo effect” of fluoridation, whereby benefits may extend to neighboring populations not formally exposed^16^. Nevertheless, ecological variables are fundamental to public policy evaluation, as they reflect the population reach and structural impacts of collective interventions aimed at reducing oral health inequalities. The lack of data regarding the duration of exposure to fluoridated water and fluoride concentrations maintained over time may have contributed to an attenuation of the protective effect on the outcomes, particularly in municipalities where the policy was recently implemented or interrupted. Despite controlling for multiple individual and contextual confounders using IPTW and multilevel models, the possibility of residual confounding by unmeasured factors remains, such as oral health-related attitudes, history of exposure to other fluoride sources, or municipal characteristics not captured by IFDM. To quantify the magnitude of the observed associations in light of this potential unmeasured confounding, E-values ​​were calculated; for the outcome of number of lost teeth, the E-value was 1.11, indicating high sensitivity to unmeasured confounding and consequently requiring caution in interpreting this result.

Although widely used, the 20-tooth cutoff for defining functional dentition does not account for the topographic distribution of missing teeth or the use of prostheses, potentially leading to an underestimation or overestimation of actual functional capacity.

The results of this study are specific to the 35-44 age group, limiting generalizability to other age groups. Tooth loss and the effects of fluoridation may manifest differently across various stages of life, justifying the need for age-specific studies.

Adults with lower levels of education and income, as well as those belonging to historically marginalized racial groups, have shown a higher risk of tooth loss^5^ ^,^ ^32^ ^,^ ^33^. While fluoridation and health care coverage are fundamental, public policies must be combined with strategies targeting vulnerable populations to effectively reduce oral health disparities.

This study reinforces that maintaining functional dentition in adults depends on universal prevention policies, such as water fluoridation and ESB coverage in primary health care, and on actions aimed at reducing social inequalities. Despite inherent limitations, particularly the cross-sectional design and the ecological measure of fluoridation, the study’s methodological robustness allowed for the identification of an association between water fluoridation and a reduction in incremental tooth loss among Brazilian adults, regardless of sociodemographic conditions.

## Supplementary Materials

**Supplementary material 1:**
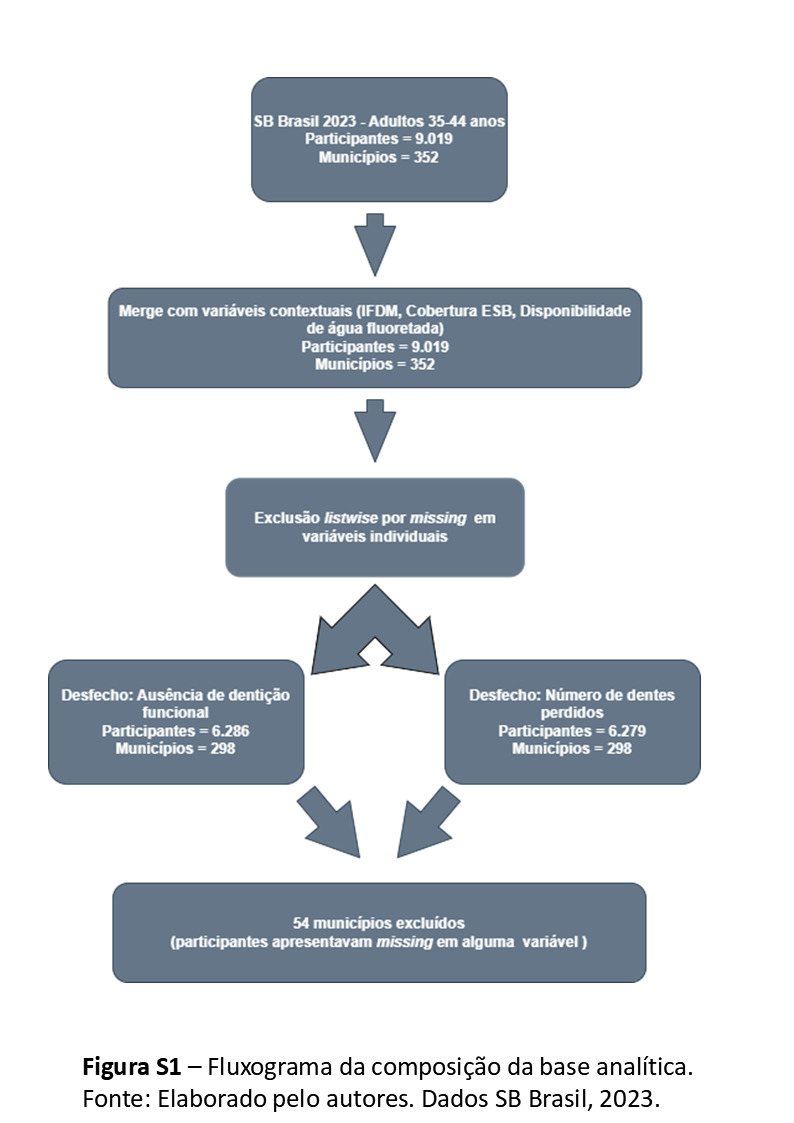
Figure 1

Supplementary material 2 Table 1
