# Supplementary material for: Water fluoridation, functional dentition and tooth loss in Brazilian adults: a multilevel analysis of SB Brasil 2023
Source: Rev Bras Epidemiol. 2026 Aug 17;29(Suppl 1):e260031supl1. doi: 10.1590/1980-549720260031.supl.1 (PMC13483508; doi:10.1590/1980-549720260031.supl.1)
Supplement: Supplementary file 1 [file 1980-5497-rbepid-29-suppl1-e260031supl1-suppl2.docx]

Com relação à redução no número de municípios que compuseram as análises multiníveis adotou-se o método de exclusão *listwise* (inclusão de casos completos) devido à proporção de dados faltantes e a opção pela não imputação de dados.

Para o desfecho ausência de dentição funcional, a exclusão de indivíduos com *missing* em qualquer variável independente resultou em 6.286 participantes distribuídos em 298 municípios, enquanto para o desfecho número de dentes perdidos, a amostra final foi de 6.279 participantes (diferença de 7 indivíduos com *missing* específico nessa variável), mantendo-se os mesmos 298 municípios. A redução de 54 municípios ocorreu porque esses municípios tinham tamanho amostral pequeno e, após a exclusão dos indivíduos com dados incompletos, nenhum residente desses municípios permaneceu elegível.

Foi testado se os 54 municípios excluídos diferiam dos 298 incluídos quanto ao número de participantes e as variáveis contextuais. Não foram observadas diferenças estatisticamente significativas entre os grupos quanto às características contextuais (IFDM, cobertura ESB e fluoretação da água) e média de participantes dos municípios dos 2 grupos, indicando que a exclusão *listwise* não introduziu viés de seleção relevante.

**Tabela suplementar (S1)**: Comparação entre número de participantes e variáveis contextuais para os municípios incluídos e excluídos das análises multinível. SB Brasil 2023.

| Variável | Municípios Incluídos (n=298) | Municípios excluídos (n=54) | Valor de p^1^ |
| --- | --- | --- | --- |
| Número de participantes, média (DP*) | 29,4 (4,2) | 4,6(0,8) | 0,993 |
| **IFDM****, n(%) |  |  |  |
| Alto | 35 (85,4) | 6 (14,6) | 0,933 |
| Médio | 133 (84,2) | 25 (15,8) |  |
| Moderado | 117 (84,8) | 21 (15,2) |  |
| Crítico | 13 (86,7) | 2 (13,3) |  |
| **Disponibilidade de água fluoretada**, n(%) |  |  | 0,616 |
| Sim | 149 (83,7) | 29 (16,3) |  |
| Não | 149 (85,6) | 25 (14,4) |  |
| **Cobertura de Saúde Bucal na AB*****, n(%) |  |  | 0,190 |
| 50% - 100% | 229 (86,1) | 37 (13,9) |  |
| < 50% | 69 (80,2) | 17 (19,8) |  |

* Desvio padrão; ** Índice FIRJAN de Desenvolvimento Municipal; *** Atenção Básica

^1^ – Valor de p do teste t de Student (para média) e qui-quadrado (para proporção).
