# Supplementary material for: Water fluoridation, functional dentition and tooth loss in Brazilian adults: a multilevel analysis of SB Brasil 2023
Source: Rev Bras Epidemiol. 2026 Aug 17;29(Suppl 1):e260031supl1. doi: 10.1590/1980-549720260031.supl.1 (PMC13483508; doi:10.1590/1980-549720260031.supl.1)
Supplement: Translation

# Translation: Fluoretação da água, dentição funcional e perda dentária em adultos brasileiros: uma análise multinível da Pesquisa Nacional de Saúde Bucal 2023

**DOI:** 10.1590/1980-549720260031.supl.1.2

**Authors:** Paulo Roberto Barbato, José Leopoldo Ferreira Antunes, Paulo Frazao, Marco Aurélio Peres

**Affiliations:** I Universidade Federal de Santa Catarina, Departamento de Fonoaudiologia - Florianópolis (SC), Brasil.; II Universidade de São Paulo, Faculdade de Saúde Pública - São Paulo (SP), Brasil.; III National Dental Research Institute Singapore, National Dental Centre - Singapore.; IV Oral Health Academic Clinical Programme, Duke-NUS Medical School - Singapore; V Health Services and Population Health Research Programme, Duke-NUS Medical School - Singapore.

**Keywords:** Perda dentária, Inquéritos, Modelagem multinível, Água fluoretada, Cuidados odontológicos

## Abstract

**Objetivo:**

Avaliar a associação entre a fluoretação da água, manutenção da dentição funcional e perdas dentárias em adultos brasileiros de 35 a 44 anos, considerando fatores individuais e contextuais.

**Métodos:**

Trata-se de estudo transversal utilizando dados da Pesquisa Nacional de Saúde Bucal (SB Brasil) 2023 (n = 9.019). A exposição principal foi a disponibilidade de água fluoretada nos municípios, obtida de sistemas de informação sobre saneamento. Os desfechos analisados foram (i) dentição funcional (binário) e (ii) número de dentes perdidos (contagem). Para reduzir viés de confusão, utilizou-se ponderação por probabilidade inversa para balancear covariáveis individuais (idade, sexo, escolaridade e renda) e contextuais (índice de desenvolvimento municipal e cobertura de serviços odontológicos). A análise foi realizada com modelos multinível de Poisson para dentição funcional e modelos multinível binomial negativa para dentes perdidos. A robustez dos resultados foi avaliada por meio de *E-values*, estimando a sensibilidade a potenciais confundidores não medidos.

**Resultados:**

Após ajuste, a fluoretação não apresentou associação significativa com dentição funcional (RP 1,24; IC95% 0,91-1,68). No entanto, associou-se significativamente a menor taxa de dentes perdidos (RT 1,30; IC95% 1,01-1,67). A análise de sensibilidade (*E-values*) indicou elevada suscetibilidade a confundidores não medidos para o desfecho de contagem.

**Conclusão:**

Em um contexto de uso disseminado de creme dental fluoretado, a fluoretação da água demonstrou efeito protetor significativo contra a perda dentária em adultos, ainda que confundida total ou parcialmente pelas variáveis socioeconômicas. Estes achados reforçam a importância de políticas universais de fluoretação, que devem ser combinadas com estratégias direcionadas a grupos vulneráveis, visando reduzir desigualdades e promover saúde bucal à população adulta.

## INTRODUÇÃO

A perda dentária permanece como um dos marcadores mais sensíveis e cumulativos das desigualdades em saúde ao longo da vida^1^ ^,^ ^2^. Embora prevenível, continua altamente prevalente entre adultos e idosos, especialmente em países de renda média, refletindo o impacto de doenças bucais não tratadas, acesso desigual aos serviços odontológicos e determinantes sociais persistentes^3^ ^,^ ^4^ ^,^ ^5^.

Este estudo analisa dois desfechos conceitualmente distintos:

1. Dentição funcional: geralmente definida por, pelo menos, 20 dentes naturais presentes associada a capacidade mastigatória, nutrição adequada e bem-estar psicossocial^6^ ^,^ ^7^, contribuindo para a qualidade de vida, o envelhecimento ativo, a redução do risco de declínio funcional e a mortalidade precoce^8^;
2. Número de dentes perdidos: desfecho cumulativo de um processo incremental e progressivo.

O primeiro representa a qualidade da dentição para desempenho de funções; o segundo, uma medida quantitativa simples.

Nas últimas duas décadas, o Brasil ampliou o acesso à saúde bucal, sobretudo com a incorporação das Equipes de Saúde Bucal (ESB) à Estratégia Saúde da Família (ESF) e expansão das políticas preventivas de base populacional^9^ ^,^ ^10^. Evidências demonstram que municípios com maior cobertura de ESF apresentam menores taxas de perda dentária e maior retenção da dentição funcional, mesmo após controle por fatores individuais e contextuais^11^ ^,^ ^12^. Persistem, porém, desigualdades regionais, por grupos de renda, escolaridade e cor/raça, indicando que acesso e uso de serviços ainda são fortemente determinados por fatores estruturais^13^.

Entre as medidas preventivas mais eficazes e equitativas, destacam-se a fluoretação das águas de abastecimento público - política que cobre cerca de 76% da população brasileira - e o uso disseminado de dentifrícios fluoretados. Ambas as medidas foram ampliadas a partir dos anos 1990^10^.

Evidências nacionais e internacionais demonstram reduções significativas na cárie, principal causa da perda dentária precoce, com maior efeito protetor da fluoretação entre grupos de menor renda^14^ ^,^ ^15^. Embora um experimento natural brasileiro tenha confirmado a fluoretação da água estar associada à redução da cárie e das perdas dentárias em adultos^16^, o efeito da fluoretação na dentição de adultos brasileiros no contexto do uso disseminado de creme dental fluoretado permanece pouco investigado. Assim, a combinação entre políticas universais (fluoretação) e estratégias de ampliação da cobertura de saúde bucal na atenção básica é fundamental para a promoção da equidade e manutenção da dentição funcional entre adultos brasileiros. Apesar de a expansão e qualificação dessas políticas não ter se completado^17^ ^,^ ^18^, investigar seus potenciais efeitos é relevante para o conhecimento científico.

Diante da complexidade dos determinantes sociais e estruturais da perda dentária, abordagens tradicionais podem subestimar efeitos causais devido ao confundimento residual e à estrutura hierárquica dos dados^19^. O uso combinado de modelos multinível e Ponderação por Probabilidade Inversa de Tratamento (IPTW) permite estimar associações entre fatores individuais, contextuais e desfechos de saúde bucal^20^ ^,^ ^21^. A avaliação por *E-values*, por sua vez, quantifica a sensibilidade a vieses residuais, fortalecendo a inferência causal em estudos observacionais^22^.

O principal objetivo deste estudo foi avaliar a associação da fluoretação da água com a manutenção da dentição funcional e perda dentária em adultos brasileiros de 35 a 44 anos, com rigoroso controle de fatores individuais e contextuais com uso de modelos multinível e ajuste por IPTW, além da quantificação da robustez das associações diante de confundidores não medidos. A hipótese principal é de que a fluoretação da água, atuando como fator de proteção populacional contra a cárie dentária, aumenta a probabilidade de retenção de dentição funcional e reduz o número de dentes perdidos.

## MÉTODOS

Estudo transversal de base populacional, com base nos dados da Pesquisa Nacional de Saúde Bucal (SB Brasil) 2023. Incluiu-se a população de 35-44 anos (9.019 participantes). A escolha desta faixa etária segue a recomendação da Organização Mundial da Saúde (OMS), permitindo comparabilidade com estudos nacionais e internacionais, incluindo as edições anteriores do SB Brasil. Foram incluídos 391 municípios para todas as idades investigadas, representando capitais e municípios do interior das 27 unidades da federação. Para adultos, os dados referem-se a residentes em 352 municípios. Detalhes amostrais e de campo constam no Relatório Final da pesquisa^23^. Os cirurgiões-dentistas examinadores foram submetidos a treinamento teórico-prático e calibração, sendo considerados aptos ao atingir concordância adequada^23^.

Este estudo utilizou dois desfechos: dentição funcional, construída com base na variável “perdido” do banco original, acrescida dos dentes perdidos por outros motivos e diminuída do total de dentes esperados em adultos, com resultado dicotomizado em “presença de dentição funcional” (20 ou mais dentes) e “ausência de dentição funcional” (menos de 20 ou nenhum dente); já o segundo desfecho foi a quantidade de dentes perdidos devido à cárie de acordo com a variável original “perdido”.

A exposição principal foi “disponibilidade de água fluoretada”, apresentada como binária (sim/não), medida no nível contextual e obtida com base no Sistema Nacional de Informações de Saneamento^24^ e Sistema de Informação de Vigilância da Qualidade da Água para Consumo Humano (anos 2022-2023)^25^. Para evitar erro de classificação, os dados de saneamento relativos a 2023 foram comparados com os dados de 2022 e 2021 e com os dados de vigilância da água para os anos de 2023 e 2022. A água de abastecimento público do município foi considerada fluoretada quando a informação foi consistente em pelo menos um dos anos considerados, em ambas as fontes de informação; ou consistente em mais de um ano considerado, em uma das fontes de informação. Os demais municípios foram considerados não fluoretados.

As covariáveis individuais incluídas foram: sexo; cor/raça; faixa etária (35-39 e 40-44 anos); anos de estudo (13 anos ou mais, 10 a 12 anos e 9 anos ou menos); renda per capita (em tercis); tempo desde a última consulta odontológica (“utilizacao_servico” no banco original) e onde consultou (“tipo_servico_utilizado” no banco original). Para o nível contextual, foi incluída também a cobertura populacional estimada pela saúde bucal na atenção básica do Sistema Único de Saúde (SUS) (proporção de habitantes potencialmente cobertos por ESB, categorizada em 50-100% e <que 50%, com base em dados do Ministério da Saúde, de dezembro de 2023)^26^. Também foi incluído o Índice Firjan de Desenvolvimento Municipal (IFDM). Este índice, que varia de 0 a 1, foi elaborado pela Federação das Indústrias do Rio de Janeiro para medir o desenvolvimento socioeconômico dos municípios brasileiros levando em conta três áreas: emprego e renda, saúde, e educação^27^. Os municípios classificam-se em: desenvolvimento alto (0,8 ou mais), moderado (0,6 a 0,79), baixo (0,4 a 0,59) e crítico (até 0,39), segundo a forma adotada pela Firjan. Os dados referem-se ao ano-base 2023 (edição de 2025).

Elaborou-se um Gráfico Acíclico Direcionado (DAG) para as perdas dentárias (Figura 1) utilizando o pacote DAGitty para o R. O DAG busca explicar como a exposição principal e as covariáveis se relacionam causalmente com os desfechos. Também explicita a estrutura hierárquica dos dados, com indivíduos aninhados em municípios. Espera-se que a fluoretação da água aumente a probabilidade de os indivíduos apresentarem dentição funcional e redução no número de dentes perdidos. Entretanto, por não se tratar de relação isolada, há a inclusão do IFDM, que é um confundidor contextual importante, pois municípios mais desenvolvidos teriam melhores condições para manter a fluoretação da água e serviços públicos odontológicos. Tais condições refletem os anos de estudo e a renda dos habitantes, que por sua vez afetam os desfechos.

**Figura 1.** Gráfico Acíclico Direcionado para as perdas dentárias em adultos. SB Brasil, 2023.

Fonte: Elaborado pelos autores. Dados SB Brasil, 2023.

Para estimar a associação da fluoretação, é necessário ajustar para o IFDM e a cobertura de ESB. O DAG também incorpora variáveis individuais proximais. A idade, forte preditor de perda dentária, reflete o acúmulo de experiência de doença ao longo da vida. As variáveis anos de estudo e renda atuam como determinantes fundamentais, influenciando os desfechos e o comportamento em saúde. Espera-se que indivíduos com maior escolaridade e renda tenham maior acesso à informação e recursos para cuidar da saúde, buscando consultas odontológicas com maior frequência e serviços que ofereçam cuidados preventivos e restauradores de maneira oportuna. O tempo desde a última consulta e tipo de serviço foram tratados como confundidores, pois antecedem potencialmente a exposição e compartilham causas comuns (nível socioeconômico), não sendo consideradas mediadoras da relação entre fluoretação e perda dentária. Não ajustar por essas variáveis poderia gerar viés de confusão residual, visto não estarem no caminho causal entre a fluoretação e perda dentária. O ajuste por esses fatores é necessário para evitar estimativas enviesadas, sem sobreajuste, refletindo o efeito direto da fluoretação, independentemente dos padrões individuais de uso de serviços.

A análise estatística foi assim organizada: descritiva com a ponderação pelos pesos amostrais, modelagem multinível para cada desfecho e análise de sensibilidade. Todas as análises foram realizadas utilizando o software *StataNow* versão 19.5. O plano amostral complexo do SB Brasil foi considerado nas análises descritivas por meio dos pesos amostrais (comando svy).

O método IPTW foi empregado para ajustar o desbalanceamento das covariáveis entre os expostos e não expostos à fluoretação da água. Escores de propensão foram estimados por regressão logística, e pesos IPTW calculados como o inverso da probabilidade de recebimento do tratamento realmente observado. Balanceamento das covariáveis entre os grupos de exposição foi verificado por meio das Diferenças Médias Padronizadas (SMD, na sigla em inglês), sendo considerado satisfatório com o valor de SMD para todas as covariáveis <0,10 na amostra ponderada.

Para os modelos multinível com IPTW, optou-se por utilizar os pesos IPTW estabilizados como *pweight*, sem incorporar os pesos amostrais complexos, pois o objetivo principal foi o balanceamento entre grupos para estimativa de associação.

As análises de regressão utilizaram estrutura de modelos multinível de dois níveis e entrada por blocos. Para “ausência de dentição funcional” foi utilizada regressão de Poisson multinível com variância robusta. A medida de efeito utilizada foi Razão de Prevalência (RP). Para “quantidade de dentes perdidos” foi utilizada regressão binomial negativa multinível para modelar o desfecho de contagem com potencial sobredispersão. Como medida de associação, foram calculadas Razões de Taxas (RT), enquanto o parâmetro de sobredispersão foi apresentado para avaliar a adequação do modelo.

Os modelos obedeceram à seguinte ordem de inclusão de variáveis: M0 (modelo vazio) - apenas o intercepto aleatório -; M1 (exposição principal); M2 (exposição principal+covariáveis individuais); e M3 (exposições contextuais+individuais).

Para avaliar os efeitos aleatórios, a magnitude da variação entre municípios foi apresentada por meio da variância do intercepto. O Coeficiente de Correlação Intraclasse (ICC, na sigla em inglês) foi calculado na análise de regressão de Poisson para quantificar a proporção da variância total do desfecho atribuível ao nível contextual. Já a razão de verossimilhança foi utilizada para comparar cada modelo com seu antecessor, verificando se a inclusão de cada novo bloco de variáveis resultava em um ajuste significativamente melhor dos dados.

Em todos os modelos de regressão foi considerada a completude das variáveis contextuais e individuais. Eventuais dados faltantes foram tratados por exclusão *listwise*. Devido à exclusão *listwise* de participantes com dados incompletos em variáveis de nível individual, a amostra final para o modelo de dentição funcional foi de 6.286 indivíduos em 298 municípios; para o modelo de número de dentes perdidos, a amostra foi de 6.279 indivíduos nos mesmos 298 municípios. Detalhes do fluxo amostral são apresentados no material suplementar (Figura S1 e Tabela S1).

Para identificar o potencial impacto de confundimento não medido, foi executada análise de sensibilidade por *E-value*, considerando a associação entre exposição principal e cada desfecho^28^. O *E-value* representa a força mínima que um confundidor não medido teria que ter para anular a associação observada entre a disponibilidade de flúor e o desfecho.

O projeto SB Brasil 2023 foi aprovado em julho de 2021, sob parecer número 4.823.054, pela Comissão Nacional de Ética em Pesquisa (Conep), de acordo com a Resolução n^o^ 466/2012 do Conselho Nacional de Saúde.

**Tabela 1.** Distribuição da população do estudo (adultos 35-44 anos), segundo desfechos e variáveis individuais e contextuais, proporção da dentição funcional e média de dentes perdidos. SB Brasil, 2023.

Desfecho	% (IC95%)*		
Dentição funcional			
Sim	92,7 (90,7-94,4)		
Não	7,3 (5,6-9,3)		
**Número de dentes perdidos**	**Média (IC95%)**	**Desvio-padrão**	
	3,5 (3,0-3,9)	5,5	
**Variáveis individuais (n=9.019)**	% (IC95%)*	**Com dentição funcional**	**Dentes perdidos**
		**% (IC95%)**	**Média (IC95%)**
Sexo			
Masculino	33,7 (31,2-36,3)	92,2 (87,8-95,0)	3,6 (2,8-4,5)
Feminino	66,3 (63,7-68,8)	93,1 (90,9-94,7)	3,4 (3,0-3,7)
Cor/raça			
Branca	42,2 (38,3-46,3)	93,9 (90,8-96,0)	2,8 (2,3-3,3)
Preta	13,5 (11,3-16,1)	92,5 (88,4-95,3)	4,3 (3,5-5,2)
Amarela	1,4 (0,9-2,1)	93,6 (84,5-97,5)	4,3 (2,8-5,8)
Parda	42,6 (38,7-46,7)	91,9 (88,6-94,3)	3,8 (3,1-4,5)
Indígena	0,3 (0,2-0,6)	92,7 (78,4-97,8)	4,7 (2,7-6,8)
Faixa etária (anos)			
35-39	49,3 (46,0-52,5)	96,1 (94,1-97,4)	2,6 (2,2-3,0)
40-44	50,7 (47,5-54,0)	89,5 (85,9-92,3)	4,3 (3,6-5,0)
Anos de estudo			
13 ou mais	23,1 (19,6-27,1)	99,4 (99,0-99,7)	1,1 (0,9-1,3)
10 a 12	48,5 (44,6-52,5)	95,2 (93,4-96,6)	2,9 (2,5-3,2)
Até 9	28,4 (25,2-31,8)	84,4 (79,7-88,1)	6,2 (5,3-7,1)
Renda per capita (em tercis)			
1º	29,5 (26,0-33,4)	87,7 (81,6-92,0)	5,2 (4,1-6,3)
2º	41,2 (36,5-46,0)	92,8 (88,1-95,7)	3,3 (2,7-3,9)
3º	29,3 (24,0-35,1)	97,6 (95,9-98,7)	2,0 (1,6-2,5)
Última consulta odontológica (anos)			
Nunca consultou	0,9 (0,5-1,7)	90,6 (65,1-98,0)	4,8 (3,0-6,7)
Até 1	51,6 (48,3-55,0)	94,1 (91,8-95,7)	3,0 (2,6-3,4)
>1-2	19,8 (17,8-22,0)	91,9 (85,1-95,7)	3,8 (2,7-4,9)
>2-3	12,6 (10,4-15,1)	89,9 (81,8-94,6)	3,7 (2,7-4,7)
>3	15,1 (12,9-17,7)	90,4 (86,2-93,4)	4,6 (3,7-5,5)
Onde consultou			
Nunca consultou	0,9 (0,5-1,7)	90,6 (65,1-98,0)	4,8 (3,0-6,7)
Serviço público	42,8 (37,5-48,4)	92,1 (89,9-93,9)	3,9 (3,4-4,5)
Serviço particular	47,0 (42,5-51,5)	91,9 (87,8-94,7)	3,3 (2,6-4,0)
Plano de saúde/convênio	8,6 (6,6-11,2)	97,7 (94,5-99,1)	2,4 (1,7-3,1)
Outros	0,7 (0,3-1,6)	91,8 (69,2-98,2)	4,0 (1,3-6,7)
Variáveis contextuais (n=352)	% (IC95%)		
IFDM			
Desenvolvimento alto	24,4 (18,4-31,7)		
Desenvolvimento moderado	50,8 (42,8-58,7)		
Desenvolvimento baixo	23,4 (19,0-28,5)		
Desenvolvimento crítico	1,4 (0,6-3,5)		
Disponibilidade de água fluoretada			
Sim	69,7 (64,3-74,5)		
Não	30,3 (25,5-35,7)		
Cobertura de saúde bucal na AB			
50-100%	54,4 (46, 5-62,0)		
<50%	45,6 (38,0-53,5)		

*Valores ponderados.

IC: intervalo de confiança; IFDM: Índice FIRJAN de Desenvolvimento Municipal; AB: atenção básica.

Fonte: Elaborado pelos autores. Dados SB Brasil, 2023.

## Declaração de disponibilidade de dados:

Todo o conjunto de dados que dá suporte aos resultados deste estudo está disponível mediante solicitação ao autor correspondente. O conjunto de dados não está publicamente disponível devido à integração de bancos de dados realizados pelos autores.

## RESULTADOS

A amostra incluiu 9.019 adultos brasileiros de 35 a 44 anos, majoritariamente mulheres (66,3%) e pardos (42,6%). A maioria apresentava dentição funcional (92,7%) e média de 3,5 dentes perdidos (DP=5,5). Quase metade tinha entre 10 e 12 anos de estudo, e 23,1% cursava ou havia concluído ensino superior. A maior parte se situava na faixa de renda per capita entre 0,36 e 0,75 salários mínimos vigentes na maior parte do ano de 2023 (R$ 1.320). Mais da metade consultou um dentista no último ano, sendo 42,8% em serviços públicos. No contexto municipal, 69,7% residiam em áreas com água fluoretada, e 54,4% em locais com cobertura de ESB superior a 50%. Predominaram municípios de desenvolvimento socioeconômico moderado (50,8%). Esses resultados refletem uma população com boas condições dentárias médias, mas ainda marcada por desigualdades estruturais de renda, escolaridade e acesso aos serviços e à fluoretação da água.

Os resultados para ausência de dentição funcional são apresentados na Tabela 2. Na análise bruta (Modelo 1), a ausência de fluoretação da água associou-se com maior prevalência do desfecho (RP 1,72; IC95% 1,22-2,43). Na análise ajustada para os fatores individuais (Modelo 2), a ausência de fluoretação associou-se a uma RP de 1,40 (IC95% 1,03-1,91). No modelo completo (Modelo 3), a RP foi atenuada para 1,24 (IC95% 0,91-1,68), com intervalo de confiança incluindo a unidade. No aspecto contextual, observou-se no modelo vazio uma expressiva variabilidade intermunicipal, com um ICC indicando que 38% da variância total na prevalência de ausência de dentição funcional é atribuível a diferenças entre municípios. Esta variabilidade reduziu-se com a inserção das covariáveis, atingindo ICC de 0,217 no modelo completamente ajustado e sugerindo que aproximadamente 43% da variância contextual inicial foi explicada pelas variáveis incluídas no modelo final.

**Tabela 2.** Regressão de Poisson com variância robusta multinível com Ponderação por Probabilidade Inversa de Tratamento para ausência de dentição funcional em adultos (35-44 anos) - n=298 municípios e 6.286 participantes. SB Brasil 2023.

Efeitos fixos	Modelo vazio	Modelo 1		Modelo 2		Modelo 3	
		RP (IC95%)*	p-valor	RP (IC95%)	p-valor	RP (IC95%)	p-valor
Fatores contextuais							
Água fluoretada							
Sim		REF.	0,002	REF.	0,031	REF.	0,141
Não		1,72 (1,22-2,43)		1,40 (1,03-1,91)		1,24 (0,91-1,68)	
Cobertura SB na AB^†^							
50-100%						REF.	0,365
<50%						0,82 (0,59-1,14)	
IFDM							
Alto						REF.	0,049
Moderado						1,48 (0,89-2,46)	
Baixo						1,76 (0,97-3,19)	
Crítico						2,98 (1,09-8,19)	
Fatores individuais							
Sexo							
Feminino				REF.	0,634	REF.	0,632
Masculino				1,11 (0,72-1,71)		1,11 (0,72-1,71)	
Cor/raça							
Branca				REF.	0,337	REF.	0,250
Preta				0,84 (0,55-1,29)		0,81 (0,52-1,24)	
Amarela				1,28 (0,75-2,18)		1,24 (0,72-212)	
Parda				0,87 (0,66-1,15)		0,84 (0,64-1,11)	
Indígena				0,89 (0,26-3,01)		0,86 (0,26-2,90)	
Faixa etária (anos)							
35-39				REF.	<0,001	REF.	<0,001
40-44				2,31 (1,78-2,99)		2,33 (1,80-3,01)	
Anos de estudo (anos)							
13 ou +				REF.	< 0,001	REF.	<0,001
10 a 12				2,34 (1,17-4,69)		2,37 (1,18-4,75)	
Até 9				5,77 (2,61-12,76))		5,85 (2,64-12,93)	
Renda per capita (tercis)							
1º				REF.	0,062	REF.	0,094
2º				0,80 (0,57-1,13)		0,83 (0,59-1,17)	
3º				0,64 (0,41-0,97)		0,66 (0,43-1,02)	
Última consulta odontológica (anos)							
Nunca consultou				REF.	0,443	REF.	0,439
Até 1				0,21 (0,02-2,21)		0,22 (0,02-2,29)	
>1-2				0,20 (0,02-2,29)		0,21 (0,02-2,35)	
>2-3				0,17 (0,02-1,73)		0,17 (0,02-1,76)	
>3				0,26 (0,03-2,63)		0,27 (0,03-2,74)	
Onde consultou							
Nunca consultou				REF.	0,280	REF.	0,351
Serviço público				6,62 (0,75-51,74)		6,39 (0,82-50,03)	
Serviço privado				6,81 (0,88-52,49)		6,67 (0,87-51,47)	
Plano saúde/convênio				4,69 (0,59-37,17)		4,58 (0,58-36,29)	
Outros				-		-	
Efeitos aleatórios							
Variância entre os municípios	0,614	0,538		0,349		0,278	
ICC	0,380	0,350		0,259		0,217	
Teste de razão de verossimilhança		p=0,013		p<0,001		p=0,058	

*Razão de prevalência e intervalo de confiança de 95%; ^†^Cobertura da saúde bucal na atenção básica.

RP: razão de prevalência; IC: intervalo de confiança; SB: saúde bucal; AB: atenção básica; IFDM: Índice FIRJAN de Desenvolvimento Municipal; IPTW: Ponderação por Probabilidade Inversa de Tratamento; ICC: Coeficiente de correlação intraclasse.

Fonte: Elaborado pelos autores. Dados SB Brasil, 2023.

A Tabela 3 apresenta os resultados da regressão binomial negativa multinível para o número de dentes perdidos. Para este desfecho, a fluoretação da água associou-se a uma menor taxa de dentes perdidos (RT 1,30; IC95% 1,01-1,67) no modelo completo, indicando que a ausência de fluoretação eleva em 30% a contagem de dentes perdidos.

**Tabela 3.** Regressão binomial negativa multinível com Ponderação por Probabilidade Inversa de Tratamento para o número de dentes perdidos em adultos (35-44 anos) - n=298 municípios e 6.279 participantes. SB Brasil 2023.

Variável	Efeitos fixos							
	Modelo 0		Modelo 1		Modelo 2		Modelo 3	
	RT (IC95%)*	p-valor	RT (IC95%)	p-valor	RT (IC95%)	p-valor	RT (IC95%)	p-valor
Água fluoretada								
Sim			REF.	0,003	REF.	0,004	REF.	0,034
Não			1,44 (1,13-1,83)		1,34 (1,09-1,64)		1,30 (1,01-1,67)	
Cobertura SB na AB^†^								
50-100%							REF.	0,557
<50%							1,05 (0,83-1,32)	
IFDM								
Alto							REF.	0,607
Moderado							1,28 (0,91-1,80)	
Baixo							1,27 (0,83-1,96)	
Crítico							1,08 (0,34-3,48)	
Sexo								
Feminino					REF.	0,246	REF.	0,246
Masculino					1,10 (0,95-1,28)		1,10 (0,95-1,28)	
Cor/raça								
Branca					REF.	0,046	REF.	0,050
Preta					1,13 (0,94-1,37)		1,13 (0,94-1,36)	
Amarela					1,24 (0,88-1,75)		1,24 (0,88-1,75)	
Parda					1,11 (1,00-1,24)		1,11 (0,99-1,24)	
Indígena					1,58 (1,04-2,39)		1,58 (1,04-2,38)	
Faixa etária (anos)								
35-39					REF.	<0,001	REF.	<0,001
40-44					1,55 (1,40-1,71)		1,55 (1,40-1,71)	
Anos de estudo (anos)								
13 ou +					REF.	<0,001	REF.	<0,001
10 a 12					1,80 (1,52-2,13)		1,81 (1,53-2,14)	
Até 9					2,74 (2,18-3,45)		2,75 (2,18-3,45)	
Renda per capita (tercis)								
1º					REF.	<0,001	REF.	<0,001
2º					0,89 (0,80-0,99)		0,89 (0,80-0,99)	
3º					0,68 (0,59-0,78)		0,68 (0,60-0,78)	
Última consulta odontológica (anos)								
Nunca consultou					REF.	0,152	REF.	0,154
Até 1					1,87 (0,64-5,48)		1,88 (0,65-5,49)	
>1-2					1,89 (0,62-5,69)		1,89 (0,63-5,69)	
>2-3					1,83 (0,63-5,31)		1,84 (0,64-5,32)	
>3					2,13 (0,69-6,54)		2,14 (0,70-6,55)	
Onde consultou								
Nunca consultou					REF.	0,663	REF.	0,666
Serviço público					0,73 (0,47-1,11)		0,72 (0,47-1,11)	
Serviço privado					0,73 (0,47-1,13)		0,73 (0,47-1,11)	
Plano saúde/convênio					0,64 (0,41-1,01)		0,64 (0,41-1,00)	
Outros					-		-	
Efeitos aleatórios								
Variância entre os municípios	0,548		0,503		0,479		0,448	
Parâmetro de sobredispersão	0,661		0,662		0,590		0,591	
Teste de razão de verossimilhança			p<0,001		p<0,001		p<0,001	

*Razão de taxas e intervalo de confiança de 95%; ^†^Cobertura da saúde bucal na atenção básica.

RT: Razão de taxas; IC: intervalo de confiança; SB: saúde bucal; AB: atenção básica; IFDM: Índice FIRJAN de Desenvolvimento Municipal.

Fonte: Elaborado pelos autores. Dados SB Brasil, 2023.

A análise dos efeitos aleatórios revelou que 18,2% da variância inicial intermunicipal foi explicada pelas covariáveis do modelo completo. O parâmetro de sobredispersão (0,591) confirmou a adequação do modelo binomial negativo, e testes de razão de verossimilhança indicaram melhora significativa no ajuste com a inclusão progressiva das covariáveis (p<0,001).

O balanceamento das covariáveis entre expostos e não expostos à água fluoretada, antes e após a aplicação dos pesos IPTW, foi calculado por meio das SMD. Antes da ponderação, algumas variáveis se apresentavam desbalanceadas; após a aplicação dos pesos IPTW, todas as covariáveis apresentaram SMD inferiores a 0,10, com maioria abaixo de 0,05, demonstrando adequado balanceamento entre os grupos. Estes resultados indicam que o procedimento de IPTW foi eficaz, criando grupos comparáveis em relação às características medidas e permitindo uma estimativa menos enviesada do efeito da fluoretação.

A análise de sensibilidade por meio dos *E-values* foi realizada para avaliar a potencial influência de confundidores não medidos. Para o desfecho dentição funcional foi encontrado um *E-value* para o limite do intervalo de confiança igual a 1,00, resultado consistente com a ausência de significância estatística observada na análise principal. Para a quantidade de dentes perdidos, o *E-value* para o limite inferior do intervalo de confiança foi de 1,11, indicando elevada suscetibilidade a confundimento residual, no qual um fator não medido de pequena magnitude poderia anular a associação observada. Portanto, uma interpretação causal deve ser cautelosa.

## DISCUSSÃO

Em adultos brasileiros de 35 a 44 anos, a disponibilidade de água fluoretada associou-se a menor número de dentes perdidos, mas não à manutenção da dentição funcional após ajuste por fatores individuais e contextuais. A associação para o desfecho contagem mostrou sensibilidade a confundimento residual, sugerindo interpretação cautelosa. Em conjunto, os achados são compatíveis com o benefício populacional da fluoretação sobre perda dentária incremental, mas não permitem inferência causal forte. Essa associação, interpretada como efeito estimado do contexto de exposição, reforça a importância das políticas públicas de saúde bucal, demonstrando que intervenções populacionais como a fluoretação da água seguem tendo impacto mensurável na preservação dos dentes funcionais.

A perda dentária em adultos brasileiros tem declinado gradualmente nas últimas décadas, resultado de políticas de prevenção e tratamento^9^. Mesmo no contexto de uso disseminado de creme dental fluoretado e de desigualdades socioeconômicas, a fluoretação da água confirmou efeito protetor sobre a perda dentária, similar ao descrito por Roberto et al.^29^

Quanto à dentição funcional, embora nas análises simples e ajustada pelas características individuais tenha sido observada associação com a fluoretação, houve perda de significância após ajuste pelo IFDM, sugerindo que o desenvolvimento municipal pode afetar as condições para implementação da fluoretação de água e manutenção da rede de serviços públicos odontológicos. A elevada prevalência de dentição funcional na amostra (92,7%) limita a capacidade de identificar fatores associados à sua ausência, podendo explicar a perda de significância da associação com a fluoretação após ajuste contextual.

Políticas universais como fluoretação podem ter seu potencial maximizado ou limitado pela dinâmica social local. Em territórios altamente desenvolvidos, duas tendências podem ser observadas: o efeito estimado geral tende a diminuir ao longo do tempo e a associação protetora tende a ser mais pronunciada em grupos socialmente vulneráveis, quando comparados aos mais afluentes^14^ ^,^ ^15^.

Os resultados reforçam a necessidade de manutenção e ampliação da fluoretação da água para prevenção da perda dentária em nível populacional, especialmente em contextos de desigualdade social. O efeito estimado da fluoretação foi consistente mesmo após ajuste para a cobertura pelas ESB, sugerindo complementaridade entre estratégia universal (fluoretação) e assistencial (ESB). Para ampliar a equidade, a expansão da fluoretação deve ser acompanhada pelo fortalecimento da atenção primária em municípios com menor desenvolvimento socioeconômico.

A interpretação isolada dos coeficientes ajustados em tabelas multiníveis - conhecida como Table 2 *fallacy* - pode gerar conclusões equivocadas sobre associação causal de variáveis individuais ou contextuais^30^. As associações observadas da fluoretação devem ser entendidas como estimativas médias condicionadas ao modelo, considerando simultaneamente interações entre fatores individuais e contextuais, e não como efeito independente absoluto do fator. A utilização de IPTW combinada com modelos multinível e análise de sensibilidade por *E-values* fortalece a inferência.

O delineamento transversal impede estabelecer relação temporal entre desfechos e exposição, embora a fluoretação da água seja uma política de longa duração e a perda dentária seja um processo cumulativo, atenuando essa limitação. Conforme discutido por Frazão e Spencer^31^, estudos observacionais sobre fluoretação da água enfrentam desafios metodológicos importantes, como a dificuldade de determinar o tempo de exposição e de controlar as variações na concentração de fluoreto durante esse tempo, podendo levar a estimativas atenuadas ou com algum grau de incerteza. Apesar disso, comparação entre expostos e não expostos com base em fontes oficiais de dados representa a melhor informação disponível utilizada em diferentes delineamentos e estudos populacionais para verificar diferenças decorrentes da exposição à fluoretação.

Outra limitação é a exposição ecológica (fluoretação), que restringe a inferência sobre efeitos individuais diretos, não permitindo identificar quem foi efetivamente exposto ou beneficiado por essa política, tampouco descartar totalmente efeitos de migração entre municípios ou efeito halo da fluoretação - que pode estender benefícios a populações vizinhas não formalmente expostas^16^. Ainda assim, variáveis ecológicas são fundamentais na avaliação de políticas públicas, pois refletem o alcance populacional e os impactos estruturais de intervenções coletivas voltadas à redução de desigualdades em saúde bucal. A indisponibilidade de informações sobre tempo de exposição à água fluoretada bem como concentração de fluoreto mantidas ao longo do tempo pode ter contribuído para atenuação do efeito protetor em relação aos desfechos, especialmente em municípios com implementação recente ou com interrupção desta política. Apesar do controle por múltiplos confundidores individuais e contextuais por meio de IPTW e modelos multinível, permanece a possibilidade de confundimento residual por fatores não medidos, como atitudes relacionadas à saúde bucal, histórico de exposição a outras fontes de flúor ou características municipais não capturadas pelo IFDM. Para quantificar a magnitude das associações encontradas diante desse potencial confundimento não medido, foram utilizados *E-values*, que para o desfecho número de dentes perdidos foi de 1,11, indicando elevada sensibilidade a confundimento não medido e, consequentemente, exigindo cautela na interpretação deste resultado.

Embora amplamente utilizado, o ponto de corte de 20 dentes para definir dentição funcional não considera a distribuição topográfica dos dentes perdidos, nem a utilização de próteses, podendo subestimar ou superestimar a real capacidade funcional.

Os resultados deste estudo são específicos para a faixa etária de 35 a 44 anos, limitando a generalização para outros grupos etários. Perdas dentárias e efeitos da fluoretação podem se manifestar de maneira distinta em diferentes fases da vida, justificando a realização de estudos específicos por faixa etária.

Adultos menos escolarizados, com menor renda e pertencentes a grupos raciais historicamente marginalizados apresentaram maior risco de perda dentária^5^ ^,^ ^32^ ^,^ ^33^. Embora fluoretação e cobertura assistencial sejam fundamentais, políticas públicas devem ser combinadas com estratégias direcionadas a populações vulneráveis para reduzir efetivamente disparidades em saúde bucal.

Este estudo reforça que a manutenção da dentição funcional em adultos depende de políticas universais de prevenção - fluoretação da água e cobertura de ESB na atenção primária - e de ações voltadas à redução das desigualdades sociais. Apesar das limitações inerentes, em especial o delineamento transversal e a medida ecológica da fluoretação, a robustez metodológica permitiu identificar associação da fluoretação da água com redução da perda dentária incremental em adultos brasileiros, independentemente das condições sociodemográficas.

## Materiais Suplementares

**Material sulplementar 1:** Figura 1

Material suplementar 2 Tabela 1
